# Empowered or Constrained? Digital Agency, Ethical Implications, and Students’ Intentions to Use Artificial Intelligence

**DOI:** 10.3390/bs16020222

**Published:** 2026-02-03

**Authors:** Dana Rad, Alina Roman, Anca Egerău, Sonia Ignat, Evelina Balaș, Tiberiu Dughi, Mușata Bocoș, Daniel Mara, Elena-Lucia Mara, Alina Costin, Radiana Marcu, Corina Costache Colareza, Claudiu Coman, Gavril Rad

**Affiliations:** 1 Center of Research Development and Innovation in Psychology, Faculty of Educational Sciences Psychology and Social Sciences, Aurel Vlaicu University of Arad, 310032 Arad, Romania; dana@xhouse.ro (D.R.); anca_petroi@yahoo.com (A.E.); soniabudean@yahoo.com (S.I.); evelinabalas@yahoo.com (E.B.); tibi_dughi@yahoo.com (T.D.); costintalina@gmail.com (A.C.); radi.marcu@yahoo.com (R.M.); 2 Faculty of Psychology and Educational Sciences, Babeş-Bolyai University of Cluj-Napoca, 400029 Cluj-Napoca, Romania; 3 Faculty of Social Sciences and Humanities, “Lucian Blaga” University of Sibiu, 550024 Sibiu, Romania; daniel.mara@ulbsibiu.ro (D.M.); lucia.mara@ulbsibiu.ro (E.-L.M.); 4 Faculty of Communication Sciences and International Relations, “Titu Maiorescu” University, 040441 Bucharest, Romania; alina.costache@prof.utm.ro; 5 Faculty of Sociology and Communication, Transilvania University of Brașov, 500036 Brașov, Romania; claudiu.coman@unitbv.ro

**Keywords:** artificial intelligence, digital agency, students, moderated mediation, perceived value, perceived benefits, intention to use AI, higher education

## Abstract

Drawing on digital agency theory, expectancy–value frameworks, and self-regulated learning perspectives, this study proposes and tests a moderated mediation model explaining students’ intentions to use AI. Using data from 673 university students, we examined whether sense of positive agency (SOPA) predicts intention to use AI indirectly through perceived value and perceived benefits of AI, and whether these pathways are conditionally shaped by sense of negative agency (SONA). Conditional process analysis (PROCESS Model 59) showed that SOPA had no direct effect on intention to use AI (b = 0.013, *p* = 0.882). Instead, its influence was fully indirect and conditional. SOPA predicted perceived value and perceived benefits of AI only at moderate to high levels of SONA, with significant SOPA × SONA interactions for both mediators (*p* = 0.040). Perceived value strongly predicted intention to use AI (b = 0.385, *p* < 0.001), and this relationship was amplified at higher levels of negative agency (b = 0.138, *p* = 0.002). In contrast, the effect of perceived benefits on intention weakened as SONA increased (b = −0.125, *p* = 0.005), becoming non-significant at higher levels of negative agency (Johnson–Neyman point ≈ 2.99). The final model explained 50.4% of the variance in intention to use AI. Overall, the findings indicate a conditional appraisal mechanism: as negative agency increases, perceived value becomes a stronger predictor of intention, whereas the motivational contribution of perceived benefits weakens and becomes non-significant beyond the Johnson–Neyman threshold. These results support an agency-aware account of AI adoption focused on how cognitive appraisals relate to intention under different perceived agency orientations, without implying ethical reasoning or moral deliberation processes not measured in this study.

## 1. Introduction

### 1.1. Artificial Intelligence in Higher Education

The integration of artificial intelligence (AI) into higher education has expanded rapidly over the past decade, reshaping teaching, learning, assessment practices, and institutional decision-making processes. Recent reviews indicate that AI has evolved from a primarily technical innovation into a strategic and pedagogical component of university systems, influencing curriculum design, learning support, academic productivity, and administrative structures (Crompton & Burke, 2023; George & Wooden, 2023; Zhou, 2023). While early debates emphasized the potentially disruptive effects of AI on academic roles and learning processes (Popenici & Kerr, 2017), more recent contributions point to a shift toward systemic and policy-level integration, particularly within innovation-oriented higher education institutions (Alqahtani & Wafula, 2025).

Across educational contexts, AI has been associated with opportunities for personalization, adaptive feedback, learning analytics, and automated academic support (Kaswan et al., 2024; Vashishth et al., 2024; Tapalova & Zhiyenbayeva, 2022). Empirical studies commonly report benefits such as increased efficiency in academic tasks, enhanced problem-solving support, individualized learning pathways, and data-informed guidance (Ahmad et al., 2022; Tang, 2024). At the same time, AI adoption in universities unfolds within broader institutional transformation processes, where pedagogical innovation intersects with governance, infrastructure, and strategic development priorities (George & Wooden, 2023; Jafari & Keykha, 2024).

Despite this expansion, a growing body of literature suggests that research on AI in higher education remains predominantly oriented toward functional, technological, and outcome-based perspectives. Systematic reviews show that much of the field has focused on mapping applications, tools, and implementation domains, often prioritizing utility, performance outcomes, or technological feasibility over psychological and behavioral mechanisms (Zawacki-Richter et al., 2019; Crompton & Burke, 2023). Even recent empirical studies on AI adoption among students and academics frequently emphasize usage frequency, perceived usefulness, or general acceptance indicators (Pisica et al., 2023; Sova et al., 2024; Grosseck et al., 2023). Research has also highlighted the importance of contextual and organizational factors in shaping AI adoption and value perceptions in educational and professional settings (Akinwalere & Ivanov, 2022).

While such approaches are essential for understanding the diffusion of AI in higher education, they may under-represent how students subjectively experience and cognitively evaluate AI use. Several studies indicate that students’ perceptions of AI are not limited to assessments of functionality or performance expectations, but are embedded in broader considerations related to trust, academic identity, self-efficacy, and perceived control over learning activities (Țală et al., 2024; Bucea-Manea-Țoniș et al., 2022). From this perspective, AI use in education can be understood not only as a pragmatic or technological behavior, but also as a psychologically mediated process involving evaluation and self-positioning in relation to technology.

Research on generative AI further supports this view, showing that students often report ambivalent attitudes that combine recognition of potential benefits with concerns regarding authorship, fairness, and reliance on automated systems (Țală et al., 2024). These findings suggest that models centered primarily on acceptance, usefulness, or performance-based outcomes may be insufficient to explain why students choose to engage with AI and under what psychological conditions such engagement becomes motivationally meaningful.

Accordingly, there is an increasing need for research that moves beyond descriptive adoption metrics and technology-centered frameworks toward behavioral and psychological perspectives that account for cognitive evaluation, motivation, and perceived agency in AI use. Responding to this need, the present study examines students’ intentions to use AI through a conditional psychological lens, focusing on digital agency and cognitive evaluation processes, and exploring how these mechanisms interact in shaping AI adoption decisions in higher education.

### 1.2. Digital Agency in Human–AI Interaction

As artificial intelligence (AI) systems become increasingly embedded in academic tasks, learning environments, and institutional processes, students’ interaction with AI extends beyond the pragmatic use of technological tools. Prior research suggests that such interactions are accompanied by shifts in how learners perceive autonomy, control, and authorship in relation to their academic activities. In educational and professional contexts, agency has been conceptualized not as a fixed individual trait, but as a subjective orientation shaped by the interaction between personal beliefs, contextual affordances, and institutional expectations (Heikonen et al., 2020). Within AI-mediated learning settings, these orientations are increasingly negotiated at the intersection of human intentionality and algorithmic mediation.

Studies on AI-supported and data-driven learning environments indicate that personalization, adaptive feedback, and automated decision support are often associated with enhanced opportunities for self-directed engagement and participation. At the same time, these same features may introduce new forms of reliance on algorithmic systems and technologically structured activity (Kaswan et al., 2024; Tapalova & Zhiyenbayeva, 2022; Vashishth et al., 2024). This dual pattern reflects broader changes in human–AI interaction, where AI systems are perceived not only as neutral tools, but as agents that influence action coordination, intention attribution, and perceived responsibility (Pagliari et al., 2022). From this perspective, AI does not merely augment cognitive performance; it alters how learners subjectively position themselves as active or constrained actors within educational activity.

Research in educational leadership and policy further highlights that AI integration has implications for institutional agency, governance, and responsibility allocation in higher education (Papa & Jackson, 2021). Such systemic developments may influence how control, authorship, and accountability are perceived to be distributed between human and technological actors in AI-mediated learning contexts (Mouta et al., 2025). Complementary findings from studies on public perceptions of AI-mediated futures reveal persistent ambivalence, characterized by simultaneous expectations of increased autonomy and concerns about loss of control or growing dependence on automated systems (Kassens-Noor et al., 2024). Together, these results suggest that autonomy in the presence of AI is often experienced as situational, negotiated, and context dependent.

Empirical research on students’ and academics’ perceptions of AI in higher education similarly points to coexisting feelings of empowerment and constraint. While perceived benefits—such as efficiency gains and learning support—are frequently reported, they are often accompanied by concerns related to dependence, erosion of skills, or blurred boundaries of authorship (Pisica et al., 2023; Țală et al., 2024; Zawacki-Richter et al., 2019). These ambivalences resonate with broader critiques in higher education governance, where tensions in perceived agency have been linked to reduced well-being in contexts dominated by externally imposed performance logics (Franco-Santos et al., 2017). In AI-mediated learning environments, such tensions may become particularly salient as students navigate shifting relations between personal initiative and algorithmic guidance.

At the same time, recent studies suggest that AI integration can also support students’ sense of agency when technologies are framed as tools for exploration, scaffolding, and self-regulated engagement, rather than as substitutes for cognitive effort (Adhikari & Pandey, 2025). Taken together, these perspectives indicate that students’ engagement with AI is more accurately understood as a psychological process of self-positioning in relation to technology, rather than as a purely instrumental adoption behavior.

Building on this reasoning, the present study conceptualizes students’ interaction with AI in terms of positive and negative agency orientations. Sense of positive agency refers to subjective perceptions of autonomy, intentionality, and control over one’s actions, whereas sense of negative agency reflects perceptions of reduced control, passivity, or external influence over outcomes. In AI-mediated learning contexts, these agency-related orientations are expected to shape how students cognitively evaluate the value and perceived benefits of AI, and how these evaluations relate to their intention to use AI. The current study advances this perspective by examining how agency-related beliefs condition the cognitive pathways through which students form intentions to use AI in higher education.

### 1.3. Cognitive Evaluation of AI: Perceived Value and Perceived Benefits

Beyond adoption and functional assessments, students’ engagement with artificial intelligence in higher education is shaped by how they cognitively evaluate the technology and the significance they attribute to its use in academic contexts. Research across technology-mediated environments indicates that individuals do not respond to AI systems solely as neutral tools, but interpret them through appraisal processes that integrate perceived usefulness, relevance, affective meaning, and trust (Shin, 2023; Wu et al., 2024). These evaluative processes underpin perceived value, a construct consistently linked to motivational readiness and behavioral intention across AI-mediated domains (J. Yin & Qiu, 2021; P. Kumar et al., 2023).

An expanding body of literature differentiates between perceived benefits and perceived value. Perceived benefits typically refer to functional, instrumental, or performance-related outcomes associated with technology use, whereas perceived value reflects a broader cognitive–motivational appraisal that incorporates judgments about importance, personal relevance, and alignment with one’s academic goals or learning orientation (Wu et al., 2024; Nguyen et al., 2025). In the context of AI use in education, perceived benefits may involve efficiency gains, feedback support, or task facilitation, while perceived value captures whether AI use is experienced as worthwhile or advantageous for one’s learning trajectory. Empirical studies suggest that, although related, these constructs may exert distinct influences on intention to engage with AI-mediated systems (Yoon & Lee, 2021; Choi et al., 2023).

Expectancy–Value Theory (EVT) provides a useful framework for understanding these evaluative mechanisms in educational settings. EVT posits that achievement-related behaviors are shaped by individuals’ expectations of success and the subjective value they assign to a task, encompassing intrinsic value, utility value, attainment value, and perceived costs (Q. Wang & Xue, 2022). Recent applications of EVT to AI-supported learning indicate that students’ intentions to engage with AI activities depend not only on competence beliefs or contextual support, but also on the value they attribute to AI as a learning resource (F. Wang et al., 2023; Lyu & Salam, 2025). Situated expectancy–value perspectives further emphasize that value appraisals are context dependent and shaped by students’ experiences within specific learning environments, including those mediated by AI technologies (S. X. Yin & Goh, 2025).

Within this theoretical framework, perceived value can be understood as a cognitive mediator between broader psychological orientations and behavioral intention. Evidence from technology adoption research shows that evaluations of value and relevance help translate beliefs and contextual affordances into motivation and subsequent intention to use technological systems (P. Kumar et al., 2023; J. Yin & Qiu, 2021). In studies focusing on generative AI, EVT-based measures demonstrate that students’ perceived value of AI predicts intention to use over and above general attitudes or perceived usefulness (Chan & Zhou, 2023). At the same time, research on AI-mediated services suggests that value appraisals may also incorporate considerations related to perceived costs or ambiguity, which can modify the strength of engagement intentions without implying explicit ethical reasoning (Nguyen et al., 2025).

These findings indicate that students’ intentions to use AI in higher education are not determined solely by pragmatic benefits or availability of technological tools, but by how AI is cognitively evaluated in relation to academic goals and learning experience. From this perspective, perceived value and perceived benefits represent distinct yet interrelated evaluative mechanisms through which students make sense of AI use. In AI-mediated learning contexts, such evaluations are likely to be shaped by students’ perceived sense of agency—that is, by the extent to which AI is experienced as supporting or constraining intentional, self-directed engagement. Building on this rationale, the present study examines perceived value and perceived benefits as cognitive mediators linking agency-related orientations to students’ intention to use AI.

### 1.4. Agency, Responsibility, and Contextual Concerns in AI Adoption

The increasing integration of artificial intelligence (AI) in educational and socio-professional environments has intensified scholarly discussions regarding autonomy, responsibility, and the redistribution of agency between human actors and intelligent systems. Contemporary research emphasizes that ethical implications of AI adoption extend beyond issues of data protection or algorithmic bias and include concerns related to decision-making authority, accountability, and cognitive participation in learning and work processes (Saurabh et al., 2022; S. Kumar et al., 2024). Importantly, ethical reasoning and moral deliberation are not measured in the present study and are not treated as explanatory mechanisms. Instead, we focus on agency-related orientations (SOPA/SONA) and on two cognitive evaluations—perceived value and perceived benefits—that can plausibly shape students’ intention to use AI. In this way, the study’s contribution is positioned as a psychological account of conditional appraisal patterns in AI adoption rather than an assessment of ethical cognition. From this standpoint, the ethical relevance of AI adoption becomes salient at the intersection between technological affordances and perceptions of who acts, who decides, and who is responsible in AI-mediated contexts.

Recent contributions in social sciences and organizational research highlight that AI systems increasingly participate in activities previously regarded as exclusively human, giving rise to hybrid configurations of action in which human and technological elements jointly influence outcomes (Symons & Abumusab, 2024). This shift raises questions not only about technical reliability, but also about perceived authorship, distribution of cognitive effort, and responsibility attribution. Ethical discussions in this area therefore focus on whether AI use is experienced as supporting intentional engagement and accountability, or as encouraging reliance on automated outputs that may attenuate personal initiative (Runcan et al., 2025).

Importantly, ethical challenges related to AI adoption are often embedded within broader processes of value creation, organizational innovation, and performance optimization. Research on AI-driven digital transformation suggests that perceived technological benefits—such as efficiency, productivity, or decision support—can dominate early evaluations of AI use, potentially delaying attention to longer-term concerns related to agency, fairness, or integrity (Saurabh et al., 2022; Maiti et al., 2025). Similarly, exploratory frameworks addressing emerging technologies indicate that AI is frequently approached initially through an instrumental lens, with more complex concerns regarding dependence, skill erosion, or redistribution of control emerging as use becomes more integrated (Serrano-Santoyo et al., 2021).

These observations align with findings from AI adoption research showing that perceived benefits and perceived value do not necessarily converge. While perceived benefits primarily reflect functional or performance-related evaluations, perceived value encompasses broader cognitive appraisals concerning relevance, significance, and coherence with one’s learning orientation. Under certain conditions, particularly when users experience tensions related to control or authorship, value-oriented evaluations may acquire increased ethical salience, not as explicit moral judgments, but as reflective appraisals of appropriateness and personal alignment.

Such considerations are particularly pertinent in educational contexts, where learning involves the development of competence, autonomy, and self-regulated engagement. When AI tools are perceived as substituting rather than supporting cognitive effort, students may experience concerns related to ownership of learning processes, authenticity of academic work, or the balance between human and automated contribution (Runcan et al., 2025). Conversely, when AI is perceived as complementing intentional engagement—by enabling exploration, scaffolding, or perspective-taking—it may be associated with more constructive forms of participation that preserve a sense of agency.

From this perspective, experiences of negative agency, such as perceived loss of control or external imposition, may be associated with more cautious or value-oriented evaluations of AI use. Importantly, such evaluations should not be interpreted as direct indicators of ethical reflection or moral deliberation, but rather as cognitive responses to perceived agency constraints. Ethical implications thus arise not from the mere presence of AI, but from how agency-related orientations shape the way AI is cognitively evaluated and integrated into academic activity.

Building on these considerations, the present study approaches ethical implications not as empirically measured outcomes or normative claims, but as interpretive consequences of the psychological patterns observed. By examining how perceived value and perceived benefits mediate the relationship between positive and negative agency orientations and students’ intention to use AI, the study contributes to a more nuanced understanding of how ethical relevance may emerge from agency-conditioned cognitive evaluations in AI adoption within higher education.

### 1.5. The Present Study and Hypotheses

Building on the theoretical perspectives outlined above, the present study examines how students’ intentions to use artificial intelligence in higher education are shaped by the interplay between agency-related beliefs and cognitive evaluations of AI. Rather than approaching AI adoption as a linear function of perceived usefulness or performance benefits, the study conceptualizes students’ engagement with AI as a process grounded in how they position themselves as agents within AI-mediated learning contexts, and how this positioning shapes the meanings and values attributed to AI use.

Specifically, we focus on two complementary forms of agency experience: sense of positive agency, reflecting perceptions of autonomy, intentionality, and control over one’s actions, and sense of negative agency, reflecting perceptions of reduced control, passivity, or external influence over outcomes. In the present study, sense of positive agency and sense of negative agency are conceptualized as subjective, self-reported orientations toward control and authorship, rather than as stable traits or direct indicators of ethical reasoning. Although these agency perceptions may be shaped by contextual experiences, the cross-sectional design of the study limits interpretation to associative relationships, rather than causal or process-based claims. In line with expectancy–value perspectives and research on cognitive appraisal in technology-mediated environments, we propose that students’ intention to use AI is not determined directly by agency-related beliefs, but rather operates through cognitive evaluation processes, namely perceived value of AI and perceived benefits of AI.

At the same time, drawing on work that highlights tensions, ambivalence, and ethical reflection in AI-mediated contexts, we assume that these cognitive pathways are not uniform across agency experiences. Instead, we expect that sense of negative agency functions as a contextual moderator, amplifying or weakening the extent to which perceived value and perceived benefits translate into intention to use AI. In other words, we examine a conditional and asymmetric psychological mechanism, in which cognitive appraisals matter differently depending on whether AI is experienced as supportive of, or intrusive upon, one’s sense of agency.

Accordingly, we anticipate that higher levels of positive agency will be associated with more favorable cognitive evaluations of AI, reflected in higher perceived value and perceived benefits, which, in turn, will be associated with stronger intention to use AI in academic contexts (H1–H3, mediation assumptions). At the same time, we expect that these indirect effects will be conditioned by negative agency experiences. We further expect that these cognitive pathways are not uniform across students. Specifically, we test whether sense of negative agency moderates (a) the associations between SOPA and the two cognitive evaluations and (b) the associations between each evaluation and intention to use AI. This conditional process perspective is strictly associative and does not imply temporal sequencing or reflective/compensatory processes; rather, it examines whether the relative predictive strength of perceived value and perceived benefits differs across levels of negative agency orientation in the present cross-sectional data (H4–H6, moderated mediation assumptions).

Finally, we expect that the direct relationship between positive agency and intention to use AI will be reduced or nonsignificant once cognitive evaluation processes are taken into account, consistent with the view that agency influences AI-use intentions primarily through evaluative and meaning-making mechanisms rather than through direct behavioral motivation (H7–H8, conditional indirect effects).

To ensure conceptual clarity and transparency, the study hypotheses are explicitly formulated and enumerated as follows:

**H1.**  *Sense of positive agency (SOPA) is positively associated with perceived value of artificial intelligence.*

**H2.**  *Sense of positive agency (SOPA) is positively associated with perceived benefits of artificial intelligence.*

**H3.**  *The relationship between sense of positive agency (SOPA) and students’ intention to use AI is mediated by perceived value and perceived benefits of AI.*

**H4.**  *Sense of negative agency (SONA) moderates the relationship between sense of positive agency (SOPA) and perceived value of AI, such that the association is stronger at higher levels of negative agency.*

**H5.**  *Sense of negative agency (SONA) moderates the relationship between sense of positive agency (SOPA) and perceived benefits of AI.*

**H6.**  *Sense of negative agency (SONA) moderates the relationships between perceived value, perceived benefits, and students’ intention to use AI.*

**H7.**  *The indirect effect of sense of positive agency (SOPA) on intention to use AI through perceived value is conditional on levels of sense of negative agency (SONA).*

**H8.**  *The indirect effect of sense of positive agency (SOPA) on intention to use AI through perceived benefits is conditional on levels of sense of negative agency (SONA).*

Taken together, the study tests a moderated mediation model in which sense of positive agency predicts students’ intention to use AI indirectly through perceived value and perceived benefits, while sense of negative agency conditions both the formation of these evaluations and their motivational impact on intention to use AI. By adopting this asymmetric conditional process perspective, the study contributes to a more nuanced understanding of the psychological mechanisms linking agency, cognitive evaluation, and AI adoption in higher education, with implications for ethically informed and agency-aware approaches to AI integration.

## 2. Materials and Methods

### 2.1. Participants and Procedure

The study employed a convenience sampling strategy and included 673 university students from Western Romania. Participants were recruited from multiple higher education institutions and study programs, encompassing diverse academic backgrounds and educational levels. The sample reflects the heterogeneous educational and occupational profiles typical of contemporary student populations in the region.

The online questionnaire was distributed to university students enrolled in higher education institutions in Western Romania through institutional social media groups associated with study programs (e.g., student group pages), alongside course-level announcements, with dissemination supported by teaching staff across study programs. To ensure eligibility, the survey introduction specified that participation was intended for currently enrolled university students in Western Romania, and respondents confirmed this criterion before proceeding. Inclusion criteria were: (a) being currently enrolled as a university student in a Western Romania higher education institution, (b) age ≥ 18 years, and (c) providing informed consent. Exclusion criteria were: (a) not meeting the student-enrollment criterion, and (b) incomplete submissions (e.g., missing responses on the focal study variables).

The gender distribution was predominantly female (87.5% women, 12.5% men). Participants’ ages ranged from 18 to 58 years (M = 29.42, SD = 10.30), indicating a mixed sample of traditional and non-traditional students. Regarding educational attainment, the largest proportions reported upper secondary education (40.6%) and bachelor’s degree completion (39.5%), followed by master’s level education (14.0%), with smaller percentages reporting postsecondary non-tertiary education, doctoral studies, or other forms of education, as seen in Table 1.

In terms of social status, most participants identified as unmarried (41.0%) or married (38.8%), while smaller proportions reported being in a stable relationship, divorced, or widowed. Occupational status varied substantially: 34.8% were employed in the public sector, 23.8% in the private sector, 33.4% reported no current employment (including students and unemployed individuals), and smaller percentages identified as entrepreneurs or freelancers.

Data were collected through an online self-report questionnaire, administered between May 2025 and June 2025. Participation was voluntary, and no financial or academic incentives were provided. Prior to completing the survey, all participants received detailed information regarding the study’s purpose and procedures and provided informed consent electronically. Responses were collected anonymously, and no identifying information was recorded. The study adhered to ethical standards for research involving human participants and complied with principles of confidentiality, anonymity, and voluntary participation. The study protocol received ethical approval from the Centre of Research Development and Innovation in Psychology of Aurel Vlaicu University of Arad (protocol code 79/15.06.2025; approval date: 15 June 2025).

### 2.2. Measures

Sense of Positive Agency was measured using the Sense of Agency Scale developed by Tapal et al. (2017). This instrument assesses individuals’ general beliefs about being the initiator and controller of their own actions, independent of specific outcomes or task success. The scale captures a dispositional sense of authorship, autonomy, and control over one’s behavior and cognitive processes. In the present study, SOPA was operationalized using the positive agency items of the original scale (e.g., items reflecting perceived control, intentionality, and self-directed action such as “I am fully in control of what I do”). Responses were recorded on a 7-point Likert scale ranging from 1 = strongly disagree to 7 = strongly agree. Item scores were averaged to compute a composite SOPA score, with higher values indicating stronger perceived positive agency. In the current sample, the SOPA subscale demonstrated good internal consistency (Cronbach’s α = 0.84).

Sense of Negative Agency was also derived from the Sense of Agency Scale (Tapal et al., 2017). This dimension captures individuals’ beliefs about reduced control, diminished authorship, or passivity in relation to their own actions, reflecting a subjective sense of being influenced or controlled by external forces. In this study, SONA was measured using the negative agency items of the original instrument (e.g., items expressing loss of control or lack of intentionality such as “My actions just happen without my intention”). Participants responded using the same 7-point Likert scale. Item scores were averaged, with higher scores indicating stronger perceptions of negative agency. The SONA subscale showed good internal reliability in the present sample (Cronbach’s α = 0.86).

Perceived value of artificial intelligence was measured using items adapted from an Expectancy–Value Theory (EVT)–based instrument assessing student perceptions of generative AI in higher education, developed by Chan and Zhou (2023). Within EVT, perceived value reflects the cognitive appraisal of a technology’s importance, usefulness, and relevance for achieving academic goals. In the present study, this construct included items assessing students’ evaluations of AI as personally meaningful and academically valuable (e.g., perceived contribution to learning quality and academic performance). Responses were provided on a 7-point Likert scale (1 = strongly disagree; 7 = strongly agree). A composite score was computed by averaging item responses. The perceived value scale demonstrated excellent internal consistency (Cronbach’s α = 0.90).

Perceived benefits of AI were assessed using items adapted from the Artificial Intelligence Attitude Scale (Aktay et al., 2024), which measures positive expectations regarding AI technologies. In line with the study’s focus, only items referring to functional and instrumental benefits were retained (e.g., increased efficiency, support for academic tasks, facilitation of idea generation). Participants rated the extent to which they perceived AI as beneficial for their academic activities on a 7-point Likert scale (1 = strongly disagree; 7 = strongly agree). Item scores were averaged to form a composite index, with higher scores indicating stronger perceived benefits of AI. The benefits scale showed high internal consistency in the present sample (Cronbach’s α = 0.88).

Students’ intention to use artificial intelligence was measured using items drawn from the same EVT-based instrument on student perceptions of generative AI (Chan & Zhou, 2023). This construct captures students’ motivational readiness and behavioral inclination to incorporate AI tools into their academic practices. Items assessed the likelihood of future AI use for learning-related purposes (e.g., coursework, assignments, or academic problem-solving). Responses were recorded on a 7-point Likert scale (1 = very unlikely; 7 = very likely). Item responses were averaged, with higher scores reflecting stronger intention to use AI. The intention scale demonstrated excellent reliability in the current study (Cronbach’s α = 0.91).

### 2.3. Data Analysis

Data analyses were conducted in IBM SPSS Statistics (v. 29) using the PROCESS macro for conditional process modeling. The analytical strategy was designed to test the hypothesized relationships between agency-related beliefs, cognitive evaluations of AI, and students’ intention to use artificial intelligence in higher education, with particular attention to the conditional nature of the indirect effects.

To address these aims, we estimated a moderated parallel mediation model (PROCESS Model 59). Sense of Positive Agency (SOPA) was specified as the independent variable, and students’ intention to use AI as the dependent variable. Perceived value of AI and perceived benefits of AI were entered as parallel mediators, consistent with the theoretical assumption that cognitive evaluations represent distinct, yet interrelated, meaning-making pathways linking agency to behavioral intention. Sense of Negative Agency (SONA) was included as a moderator on both the a-paths (from SOPA to each mediator) and the b-paths (from mediators to intention to use AI), allowing examination of whether agency tensions condition both the formation of cognitive evaluations and their motivational impact on intention.

Indirect and conditional indirect effects were estimated using bias-corrected bootstrapping with 5000 resamples, which provides robust confidence intervals without relying on normality assumptions. Effects were considered statistically significant when the 95% bootstrap confidence interval did not include zero. The decision to adopt a bootstrapped conditional process approach reflects the study’s underlying premise that relationships among agency, evaluation, and intention are likely to be asymmetric and context dependent, rather than uniform across students.

To further interpret significant moderation effects, we computed simple slopes at low, moderate, and high levels of the moderator (±1 SD) and applied the Johnson–Neyman technique, which identifies the range of SONA values for which the predictor–outcome relationship becomes statistically significant. This approach enabled a more precise examination of how negative agency experiences delimit the conditions under which perceived value and perceived benefits meaningfully predict intention to use AI.

All analyses were performed using two-tailed tests with a significance threshold of *p* < 0.05. Prior to estimation, assumptions regarding normality, multicollinearity, and outliers were screened, and descriptive statistics and Pearson correlations were computed to contextualize the conditional process estimates.

## 3. Results

### 3.1. Descriptive Statistics and Correlations

Descriptive statistics and zero-order correlations among the study variables are presented in Table 2. Overall, students reported a relatively high level of Sense of Positive Agency (SOPA), with a mean above the midpoint of the scale (M = 5.85, SD = 0.98), whereas Sense of Negative Agency (SONA) was comparatively low (M = 2.40, SD = 1.00), suggesting that most students tended to perceive themselves as intentional and self-directed rather than passive or externally constrained in their actions.

With respect to AI-related cognitive evaluations, students reported moderately positive perceptions of the perceived benefits of AI (M = 3.68, SD = 0.90) and perceived value of AI (M = 3.45, SD = 0.90). The mean level of intention to use AI was somewhat lower (M = 3.08, SD = 1.07), indicating a more cautious orientation toward future engagement with AI tools.

Consistent with the theoretical expectations, SOPA showed positive correlations with perceived value, perceived benefits, and intention to use AI, whereas SONA correlated negatively with these outcomes. Perceived value and perceived benefits were moderately and positively correlated with one another and with students’ intention to use AI, supporting their role as conceptually related yet distinct cognitive predictors of behavioral intention. These associations provided initial justification for testing the proposed moderated parallel mediation model.

The intercorrelations among the study variables are presented in Table 3. As expected, Sense of Positive Agency (SOPA) and Sense of Negative Agency (SONA) were strongly and negatively correlated (r = −0.43, *p* < 0.001), indicating that students who perceived themselves as more intentional and self-directed tended to report fewer experiences of reduced or externally constrained agency.

SOPA was weakly but positively correlated with perceived benefits of AI (r = 0.12, *p* < 0.01), suggesting that students with higher positive agency evaluated AI as slightly more beneficial. In contrast, SOPA was not significantly correlated with perceived value of AI (r = −0.02, *p* = ns) or with intention to use AI (r = −0.01, *p* = ns), indicating that agency beliefs did not translate directly into stronger behavioral intention.

Conversely, SONA showed a small but positive correlation with perceived value of AI (r = 0.09, *p* < 0.05) and with intention to use AI (r = 0.12, *p* < 0.01), suggesting that students who reported greater negative agency experiences were also somewhat more inclined to attribute value to AI and to express intention to use it. This pattern is consistent with the theoretical assumption suggesting that higher negative agency orientation co-occurs with slightly higher perceived value and intention in this sample, without implying compensatory processes beyond the observed associations.

Perceived value and perceived benefits of AI were moderately and positively correlated (r = 0.60, *p* < 0.001), supporting their conceptual relatedness while indicating that they represent distinguishable evaluative constructs. Both variables showed substantial positive associations with intention to use AI, with a particularly strong effect for perceived value (r = 0.69, *p* < 0.001) compared to perceived benefits (r = 0.51, *p* < 0.001). These results support the premise that cognitive evaluations play a central role in motivating AI-use intentions.

The correlational findings provide preliminary evidence consistent with the hypothesized cognitive mediation mechanism, while also supporting the rationale for examining whether these relationships operate differentially across levels of negative agency through moderated mediation analyses.

### 3.2. Effects of SOPA on the Mediators

Consistent with the hypotheses formulated in Section 1.5, we tested a moderated parallel mediation model examining the direct, indirect, and conditional effects of digital agency on students’ intention to use artificial intelligence.

**Figure 1:**
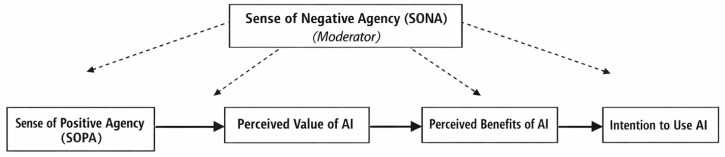
Conceptual moderated mediation model linking digital agency to students’ intention to use artificial intelligence.

**Figure 2:**
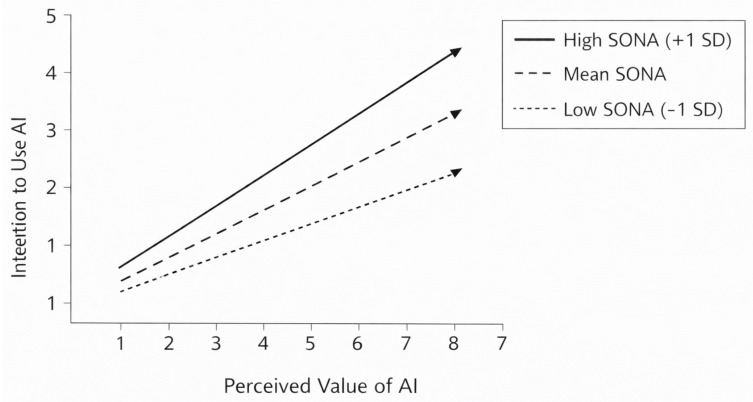
Interaction between perceived value of AI and sense of negative agency (SONA) predicting intention to use AI.

**Figure 3:**
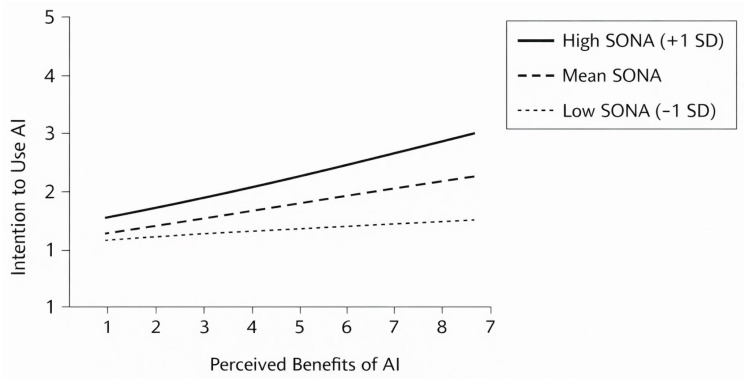
Interaction between perceived benefits of AI and sense of negative agency (SONA) predicting intention to use AI.

In addition to the tabular results, we provide visual summaries of the conceptual model and the key interaction effects (Figure 1, Figure 2 and Figure 3) to improve interpretability.

The conceptual model (Figure 1) illustrates a moderated parallel mediation framework in which Sense of Positive Agency (SOPA) predicts students’ intention to use artificial intelligence indirectly through two cognitive evaluation pathways: perceived value of AI and perceived benefits of AI. Sense of Negative Agency (SONA) functions as a contextual moderator, conditioning both the paths from SOPA to the mediators and the paths from the mediators to intention to use AI. The model reflects the hypothesized asymmetric and conditional mechanisms tested in the study.

The first step of the conditional process analysis examined whether Sense of Positive Agency (SOPA) predicted students’ perceived value of AI and perceived benefits of AI, and whether these effects varied as a function of Sense of Negative Agency (SONA). The results indicated statistically significant SOPA × SONA interactions for both mediators, supporting the assumption that cognitive evaluations of AI vary systematically across levels of perceived agency tension rather than uniformly across students. Results are presented in Table 4.

The model predicting perceived value of AI was significant (F = 3.58, *p* = 0.014, R^2^ = 0.016), and the SOPA × SONA interaction reached statistical significance (b = 0.0736, *p* = 0.040). This indicates that the relationship between positive agency and perceived value tends to be relatively more influential by the level of negative agency. Simple-slope analyses showed that: at low SONA (−1 SD), SOPA was not significantly related to perceived value (b = −0.07, *p* = 0.25), at mean SONA, SOPA remained unrelated (b = 0.00, *p* = 0.93), at high SONA (+1 SD), SOPA became positively associated with perceived value (b = 0.08, *p* = 0.096; marginal trend).

The Johnson–Neyman test identified a transition point at SONA ≥ 3.89, above which the effect of SOPA on perceived value became statistically significant (*p* ≤ 0.050).

This pattern suggests that students who experience stronger negative agency are more likely to cognitively valorize AI when they also report high positive agency, meaning that AI may be appraised as meaningful particularly in contexts where students feel simultaneously constrained and agentic. In other words, positive agency predicts perceived value only under high perceived loss of control, consistent with an interpretive pattern indicating that the association is stronger under higher negative agency orientation, consistent with a conditional appraisal pattern rather than a compensatory or reflective process.

A similar pattern emerged for perceived benefits of AI (F = 4.63, *p* = 0.003, R^2^ = 0.020), with a significant SOPA × SONA interaction (b = 0.0731, *p* = 0.040).

Conditional effects indicated that at low SONA (−1 SD), SOPA had no effect on perceived benefits (b = 0.02, *p* = 0.70), at mean SONA, SOPA significantly predicted higher perceived benefits (b = 0.10, *p* = 0.017), at high SONA (+1 SD), the effect strengthened further (b = 0.17, *p* < 0.001).

The Johnson–Neyman region showed that the SOPA effect became significant from SONA ≥ 2.22, meaning that even moderate levels of negative agency were sufficient for positive agency to predict increased perceived benefits.

When students report a stronger sense of constraint or loss of control, higher positive agency is associated with stronger instrumental appreciation of AI, suggesting that AI is perceived as a functional resource for regaining control or managing academic demands. Compared with perceived value, perceived benefits appear to be activated earlier along the SONA continuum, indicating that utilitarian rather than identity-relevant meaning may be the first pathway through which agency tension shapes AI evaluation.

### 3.3. Effects of Mediators on Intention to Use AI

The second step of the model examined whether perceived value of AI and perceived benefits of AI predicted students’ intention to use AI, and whether these relationships varied across levels of Sense of Negative Agency (SONA). The results revealed a differential moderation pattern, indicating that the motivational relevance of the two cognitive evaluative pathways operates asymmetrically under conditions of negative agency. Results are presented in Table 5.

The model predicting intention to use AI based on perceived value was significant, and the Perceived Value × SONA interaction reached statistical significance, indicating that the motivational impact of perceived value varies as a function of students’ negative agency levels.

Simple slope analyses showed that at low SONA (−1 SD), perceived value was positively related to intention b = 0.44, *p* < 0.001, at mean SONA, the effect increased in magnitude b = 0.55, *p* < 0.001, at high SONA (+1 SD), the relationship became strongest b = 0.66, *p* < 0.001.

The Johnson–Neyman procedure indicated that the effect of perceived value on intention to use AI was significant across the entire observed range of SONA, but increased progressively with higher levels of negative agency.

The positive association between perceived value of AI and intention to use AI becomes stronger as levels of negative agency increase, indicating a value-driven motivational mechanism under conditions of reduced perceived control (Figure 2).

When students report higher levels of negative agency, perceived value is more strongly associated with intention to use AI, suggesting that AI tends to be appraised as more meaningful in contexts characterized by lower perceived control. This finding supports an interpretation according to which value-based engagement with AI may become more salient under conditions of reduced perceived control, consistent with an interpretive pattern indicating that the association is stronger under higher negative agency orientation, consistent with a conditional appraisal pattern rather than a compensatory or reflective process.

A contrasting pattern emerged for perceived benefits of AI. Although perceived benefits predicted intention to use AI at lower levels of SONA, the Benefits × SONA interaction was negative, indicating that the motivational relevance of benefits decreased as negative agency increased.

Conditional effects showed that at low SONA (−1 SD), benefits significantly predicted intention b = 0.38, *p* < 0.001, at mean SONA, the effect weakened b = 0.25, *p* < 0.01, at high SONA (+1 SD) the relationship became non-significant b = 0.08, *p* = ns.

The Johnson–Neyman region revealed that the effect of perceived benefits ceased to be significant at approximately SONA ≥ 3.10, indicating that under stronger negative agency, functional or performance-based justifications no longer translate into behavioral intention.

As illustrated in Figure 3, the positive association between perceived benefits of AI and intention to use AI weakens as sense of negative agency increases, becoming negligible at higher levels of perceived loss of control.

Under conditions of reduced control, instrumental appreciation of AI loses motivational significance, whereas value-based meaning becomes the dominant driver of intention. This pattern supports the theorized asymmetric cognitive pathway: benefits motivate use when students feel agentic and unconstrained and value motivates use when agency is threatened.

When students feel agentic and in control, intention is more strongly associated with instrumental benefits of AI and when students experience constraint or loss of control, intention tends to be relatively more influential than perceived value and meaning of AI use. This provides strong support for the study’s central claim that AI adoption in higher education is not a purely functional decision, but a context-dependent cognitive–motivational process shaped by agency experiences.

### 3.4. Direct and Conditional Indirect Effects

The full conditional process model predicting students’ intention to use AI was statistically significant, explaining a substantial proportion of variance (R^2^ = 0.504). Importantly, the direct effect of SOPA on intention to use AI was not significant (b = −0.05, *p* = ns), indicating that positive agency does not exert a direct motivational influence on AI-use intentions once cognitive evaluations are taken into account. This pattern is consistent with the theoretical assumption that agency beliefs influence behavioral intention indirectly, through evaluative meaning-making processes. Results are presented in Table 6.

The indirect effect of SOPA on intention via perceived value of AI was small and nonsignificant at low SONA (b = 0.01, 95% CI: −0.01; 0.05), moderate and significant at mean SONA (b = 0.05, 95% CI: 0.01; 0.10), strongest and clearly significant at high SONA (b = 0.09, 95% CI: 0.03; 0.18). Thus, the value-based pathway becomes more influential as negative agency increases.

When students experience higher levels of negative agency, positive agency contributes to AI-use intentions primarily by strengthening the perceived value and meaningfulness of AI. This pattern is consistent with a conditional appraisal account in which the association between perceived value and intention strengthens as negative agency increases, without implying compensatory or reflective processes beyond the observed moderation effects.

In contrast, the indirect effect via perceived benefits of AI followed the opposite trend, significant at low SONA (b = 0.06, 95% CI: 0.02; 0.13), reduced at mean SONA (b = 0.04, 95% CI: 0.01; 0.09), nonsignificant at high SONA (b = 0.01, 95% CI: −0.02; 0.06). Thus, the benefits-based pathway loses motivational relevance when negative agency increases.

When students feel constrained or less in control, instrumental benefits are no longer sufficient to motivate AI use, and intention tends to be more strongly associated with perceived value. The contrast between the two mediators provides evidence consistent with an asymmetric conditional mediation pattern.

The final moderated mediation model accounted for 50.4% of the variance in students’ intention to use AI, indicating a strong overall explanatory capacity. Taken together, the results converge toward a coherent psychological mechanism in which positive agency does not exert a direct effect on intention to use AI, but instead influences behavioral motivation indirectly, through cognitive appraisal processes. The findings show that the two evaluative pathways, perceived benefits and perceived value are activated asymmetrically across levels of negative agency. When students report lower levels of negative agency and experience their learning context as relatively unconstrained, intention to use AI is primarily explained by perceived instrumental benefits, such as efficiency and task support. By contrast, when students experience higher levels of negative agency and perceive a diminished sense of control or authorship, intention to use AI is driven predominantly by perceived value and meaning, indicating a moderation-dependent shift in the relative motivational relevance of perceived value versus perceived benefits across levels of negative agency.

From an expectancy–value perspective, this pattern indicates that perceived value (i.e., meaning and goal relevance) becomes especially salient under agency tension, consistent with EVT-based operationalizations of students’ AI appraisals in higher education (Chan & Zhou, 2023).

Under conditions of heightened agency tension, perceived benefits lose much of their motivational relevance, whereas perceived value becomes the dominant mechanism through which positive agency contributes to AI-use intention. This pattern suggests that students do not adopt AI merely because it is perceived as useful, but rather because it acquires personal and academic significance in situations where their sense of agency is challenged or destabilized. Overall, the findings underscore the importance of approaching AI integration in higher education from an agency-aware perspective, recognizing that students’ engagement with AI is shaped not only by functional utility but also by deeper processes of self-positioning, authorship, and control in learning.

## 4. Discussion and Implications

The findings of the present study indicate that students’ engagement with artificial intelligence in higher education is best understood as a conditional, agency-dependent evaluative process, rather than as a purely functional or utility-driven adoption mechanism. While prior research has highlighted the transformative potential of AI for personalized learning, feedback systems, and academic efficiency, it has also documented tensions related to autonomy, responsibility, and the reconfiguration of learning processes in AI-mediated environments (Zaharia, 2024; Sîrghi et al., 2024; Marian et al., 2023). The current results extend this literature by showing that sense of positive agency alone does not directly predict students’ intention to use AI. Instead, its influence is expressed indirectly through cognitive evaluation pathways whose motivational relevance varies as a function of negative agency. This pattern suggests that students’ evaluations of AI are shaped not simply by general confidence or self-directedness, but by how agency-related orientations interact with perceived constraints in academic contexts.

The asymmetric structure of the cognitive mechanisms observed in this study is particularly informative. When negative agency is low and students experience their learning environment as manageable and self-directed, intention to use AI is explained primarily by perceived instrumental benefits, consistent with perspectives that frame AI as a complementary learning aid or performance-support tool (Fortea et al., 2025; Mara et al., 2024). By contrast, as negative agency increases and students report lower perceived control or authorship, the motivational influence of perceived benefits weakens, while perceived value becomes the dominant pathway linking positive agency to AI-use intention. In such contexts, AI appears to acquire significance not because of efficiency gains, but because it is cognitively appraised as relevant or meaningful for one’s academic engagement. This shift is consistent with findings in educational psychology indicating that value-based evaluation becomes more salient under conditions of uncertainty, constraint, or challenge (Stavropoulou & Stamovlasis, 2024).

From a theoretical perspective, these results refine classical technology acceptance models by demonstrating that intentions toward AI cannot be reduced to utilitarian benefit appraisal alone. Rather, they reflect a dual evaluative architecture, in which perceived benefits are more influential when agency remains relatively stable, whereas perceived value becomes decisive when agency-related constraints are salient. Similar dynamics have been reported in studies examining motivational engagement and AI-related beliefs in educational contexts, where value-oriented constructs mediate complex relationships between motivation, technology use, and self-regulative functioning (Rad & Roman, 2025; Rad et al., 2024). In this sense, the present findings show that perceived value does not merely coexist with perceived benefits, but may assume a primary motivational role under specific agency conditions, indicating an adaptive cognitive response rather than a purely instrumental orientation toward AI.

From an ethical standpoint, the relevance of these findings lies not in demonstrating ethical reasoning as an empirical process, but in highlighting how agency-related orientations may shape the ethical salience of AI use in educational contexts. When intention to use AI is driven predominantly by perceived benefits, engagement may remain efficiency-oriented and instrumental, a concern frequently raised in discussions on ethical AI use in academic environments (Lazăr et al., 2024). In contrast, the value-dominant pattern observed under higher levels of negative agency suggests that students may evaluate AI in relation to broader considerations of authorship, responsibility, and personal alignment, rather than utility alone.

Importantly, ethical reasoning or moral deliberation were not directly measured in the present study. The observed patterns should therefore not be interpreted as evidence of ethical reflection per se, but as cognitive evaluation processes that may acquire ethical relevance under conditions of perceived agency constraint. This interpretation is consistent with ongoing discussions on cognitive substitution, redistribution of responsibility, and the shifting boundaries between human judgment and algorithmic mediation in higher education (Zaharia, 2024; Sîrghi et al., 2024). Accordingly, ethical implications are best understood here as interpretive consequences of how students cognitively evaluate AI in relation to their sense of control and authorship, rather than as empirically demonstrated moral processes.

At the level of educational practice, these findings suggest that AI integration strategies should move beyond a narrow focus on technical competence or productivity enhancement. Instead, they should support students’ capacity for intentional, self-directed engagement in AI-rich learning environments. Research on smart learning ecosystems, digital transformation, and competence-oriented curriculum reform emphasizes the importance of fostering both technological fluency and agency-aware learning designs to prevent passive or compensatory reliance on automated systems (Fortea et al., 2025; Marian et al., 2023; Mara et al., 2024). From this perspective, AI literacy initiatives may benefit from emphasizing when, why, and under what conditions AI becomes valuable for learning, rather than focusing exclusively on functional benefits. Such approaches align with evidence showing that motivational architectures related to technology engagement are multidimensional and embedded in self-regulatory and meaning-making processes (Rad & Roman, 2025).

Several limitations of the present study should be acknowledged. First, the cross-sectional design precludes causal inference and does not allow conclusions regarding the temporal sequencing of agency orientations and cognitive evaluations. Longitudinal research could clarify whether value-oriented appraisals emerge as stable motivational patterns or as situational responses to specific learning demands. Second, reliance on self-report measures may introduce biases related to social desirability or subjective interpretation. Future studies incorporating behavioral indicators of AI engagement or learning outcomes would enhance ecological validity. Third, the study was conducted within a specific cultural and institutional context, which may limit generalizability. Comparative or cross-cultural research could examine whether the asymmetric evaluative pathways identified here are robust across different educational systems and normative environments.

Future research may extend the present conditional process framework using longitudinal or multi-wave designs, or alternative analytical approaches such as structural equation modeling, to capture reciprocal dynamics between agency-related orientations, cognitive evaluation, and AI-use behavior. Such work would contribute to a more comprehensive understanding of how students construct meaning, allocate cognitive effort, and negotiate responsibility in increasingly AI-mediated learning environments, advancing both theoretical and ethically informed perspectives on AI integration in higher education.

## 5. Conclusions

The present study advances current understanding of AI adoption in higher education by demonstrating that students’ intention to use artificial intelligence is not driven solely by instrumental or performance-oriented evaluations, but emerges from a conditional psychological mechanism shaped by agency-related orientations. The moderated mediation results indicate that sense of positive agency does not exert a direct influence on intention to use AI; instead, its effects operate indirectly through cognitive appraisal pathways whose motivational relevance varies depending on levels of negative agency. When agency remains relatively stable, intention to use AI is primarily associated with perceived benefits, reflecting an instrumental and efficiency-oriented orientation toward technology. By contrast, as negative agency intensifies and students experience reduced control or authorship, the evaluative structure shifts, with perceived value—rather than perceived benefits—becoming the dominant pathway through which AI acquires motivational significance.

These findings extend prior conceptual work on human agency in AI-mediated contexts by showing that agency-related orientations are not merely background dispositions, but play a central role in shaping how students translate cognitive appraisals of AI into adoption intentions. In line with agency-based perspectives, the results underscore the importance of perceived control and authorship for understanding technology engagement in learning environments (e.g., Tapal et al., 2017; Pagliari et al., 2022; Papa & Jackson, 2021).

The asymmetric evaluative mechanism identified in this study represents its primary theoretical contribution. It suggests that AI engagement in academic contexts cannot be adequately captured by linear or exclusively benefit-driven acceptance models. Instead, it requires an agency-aware framework in which perceived benefits and perceived value function differently depending on students’ agency-related experiences. From this perspective, AI use is not simply a response to functional affordances, but is integrated into students’ learning activity through cognitive evaluations that reflect how technology is positioned in relation to control, authorship, and academic engagement.

Although ethical debates are often discussed in parallel with agency and authorship in AI-mediated learning, the present study did not operationalize ethical reasoning, moral deliberation, or ethical decision-making. Accordingly, the findings should be interpreted as evidence of a conditional psychological mechanism linking agency orientations, cognitive appraisals (value/benefits), and intention to use AI. Any ethics-related considerations are therefore outside the inferential scope of the present dataset and are offered only as contextual motivation for future research that directly measures ethical cognition and behavior.

Accordingly, the study supports an understanding of AI adoption in higher education as an agency-aware decision, rather than a passive or norm-driven trajectory of technological uptake. Designing educational environments that support intentional, self-directed engagement with AI—especially under conditions where students report higher negative agency—may foster more sustainable and pedagogically aligned use of AI tools. Thus, the present study does not assess ethical reasoning or moral deliberation. Its contribution is therefore best understood as psychological: it identifies an agency-conditioned pattern in which perceived value and perceived benefits show different motivational relevance for intention to use AI across levels of negative agency orientation. Future studies that directly operationalize ethical cognition and AI-use behaviors could test whether, and under what conditions, such agency-conditioned appraisal patterns relate to ethical decision-making in educational settings.

## Figures and Tables

**Table 1:** Sociodemographic characteristics of the sample (N = 673).

Variable	Category	n	%
**Gender**	Female	589	87.5
	Male	84	12.5
**Age (years)**	Range	18–58	—
	Mean (SD)	29.42 (10.30)	—
**Educational Level**	Upper secondary (high school)	273	40.6
	Bachelor’s degree	266	39.5
	Master’s degree	94	14.0
	Postsecondary non-tertiary	30	4.5
	Doctoral studies	6	0.9
	Other forms of education	4	0.6
**Social Status**	Unmarried	276	41.0
	Married	261	38.8
	In a stable relationship	102	15.2
	Divorced	29	4.3
	Widowed	5	0.7
**Occupational Status**	Public sector employee	234	34.8
	Private sector employee	160	23.8
	Not employed (student, unemployed, retired)	225	33.4
	Entrepreneur/business owner	36	5.3
	Freelancer/self-employed	18	2.7

**Table 2:** Descriptive statistics for study variables (N = 673).

Variable	Min	Max	M	SD
Sense of Positive Agency (SOPA)	2.00	7.00	5.85	0.98
Sense of Negative Agency (SONA)	1.00	7.00	2.40	1.00
Perceived Benefits of AI	1.00	5.00	3.68	0.90
Perceived Value of AI	1.00	5.00	3.45	0.90
Intention to Use AI	1.00	5.00	3.08	1.07

Note. M = Mean; SD = Standard Deviation.

**Table 3:** Pearson correlations results.

Variable		SOPA	SONA	Benefits of Artificial Intelligence	Student Perceived Value of AI	Students Intention to Use AI
1. SOPA	Pearson’s r	-				
2. SONA	Pearson’s r	−0.430 ***	-			
3. Benefits of Artificial Intelligence	Pearson’s r	0.115 **	−0.021	-		
4. Student Perceived Value of AI	Pearson’s r	−0.018	0.094 *	0.600 ***	-	
5. Students Intention to Use AI	Pearson’s r	−0.014	0.123 **	0.514 ***	0.689 ***	-

* *p* < 0.05, ** *p* < 0.01, *** *p* < 0.001.

**Table 4:** Moderated effects of Sense of Positive Agency (SOPA) on perceived value and perceived benefits of AI as a function of Sense of Negative Agency (SONA).

Outcome	Predictor	b	SE	t	*p*	95% CI
Perceived Value of AI	SOPA	−0.17	0.10	−1.66	0.097	−0.38; 0.03
	SONA	−0.34	0.21	−1.58	0.115	−0.76; 0.08
	SOPA × SONA	0.07	0.04	2.06	0.040	0.00; 0.14
Perceived Benefits of AI	SOPA	−0.08	0.10	−0.76	0.445	−0.28; 0.12
	SONA	−0.40	0.21	−1.88	0.060	−0.82; 0.02
	SOPA × SONA	0.07	0.04	2.06	0.040	0.00; 0.14

**Table 5:** Moderated effects of perceived value and perceived benefits of AI on intention to use AI as a function of Sense of Negative Agency (SONA).

Predictor	b	SE	t	*p*	95% CI
Perceived Value of AI	0.55	0.04	13.75	<0.001	0.47; 0.63
Value × SONA	0.08	0.03	2.62	0.009	0.02; 0.14
Perceived Benefits of AI	0.25	0.07	3.57	<0.001	0.11; 0.39
Benefits × SONA	−0.12	0.05	−2.39	0.017	−0.21; −0.02

**Table 6:** Direct and conditional indirect effects of SOPA on intention to use AI across levels of SONA.

Effect	SONA Level	b	Boot SE	95% CI (LLCI–ULCI)	Sig.
Direct effect (SOPA → Intention)	-	−0.05	0.06	−0.17–0.06	ns
Indirect via Perceived Value	Low (−1 SD)	0.01	0.02	−0.01–0.05	ns
	Mean	0.05	0.02	0.01–0.10	✓
	High (+1 SD)	0.09	0.03	0.03–0.18	✓
Indirect via Perceived Benefits	Low (−1 SD)	0.06	0.03	0.02–0.13	✓
	Mean	0.04	0.02	0.01–0.09	✓
	High (+1 SD)	0.01	0.02	−0.02–0.06	ns

ns—not significant and ✓—significant.

## Data Availability

Dataset generated and analyzed during the current research is available upon reasonable request from the corresponding author.
